# Preparation and Effect of Methyl-Oleate-Based Polyol on the Properties of Rigid Polyurethane Foams as Potential Thermal Insulation Material

**DOI:** 10.3390/polym15143028

**Published:** 2023-07-13

**Authors:** Norsuhaili Kamairudin, Luqman Chuah Abdullah, Seng Soi Hoong, Dayang Radiah Awang Biak, Hidayah Ariffin

**Affiliations:** 1 Higher Education Centre of Excellence (HiCoE), Institute of Tropical Forestry and Forest Product, University Putra Malaysia, Serdang 43400, Selangor, Malaysia; hidayah@upm.edu.my; 2 Department of Chemical and Environmental Engineering, Faculty of Engineering, University Putra Malaysia, Serdang 43400, Selangor, Malaysia; dradiah@upm.edu.my; 3 Malaysian Palm Oil Board, No. 6, Persiaran Institusi, Bandar Baru Bangi, Kajang 43000, Selangor, Malaysia; sengsoi@mpob.gov.my; 4 Institute of Nanoscience and Nanotechnology, Universiti Putra Malaysia, Serdang 43400, Selangor, Malaysia; 5 Department of Bioprocess Technology, Faculty of Biotechnology and Biomolecular Sciences, Universiti Putra Malaysia, Serdang 43400, Selangor, Malaysia

**Keywords:** amidation reaction, alkanolamide polyol, renewable material, rigid polyurethane foams, thermal conductivity

## Abstract

Recently, most of the commercial polyols used in the production of rigid polyurethane foams (RPUFs) have been derived from petrochemicals. Therefore, the introduction of modified palm oil derivatives-based polyol as a renewable material into the formulation of RPUFs is the focus of this study. A palm oil derivative—namely, methyl oleate (MO)—was successfully modified through three steps of reactions: epoxidation reaction, ring-opened with glycerol, followed by amidation reaction to produce a bio-based polyol named alkanolamide polyol. Physicochemical properties of the alkanolamide polyol were analyzed. The hydroxyl value of alkanolamide polyol was 313 mg KOH/g, which is suitable for producing RPUFs. Therefore, RPUFs were produced by replacing petrochemical polyol with alkanolamide polyol. The effects of alkanolamide polyol on the physical, mechanical and thermal properties were evaluated. The results showed that the apparent density and compressive strength increased, and cell size decreased, upon introducing alkanolamide polyol. All the RPUFs exhibited low water absorption and excellent dimensional stability. The RPUFs made with increased amounts of alkanolamide polyol showed higher thermal conductivity. Nevertheless, the thermal conductivities of RPUFs made with alkanolamide polyol are still within the range for thermal insulating materials (<0.1 W/m.K). The thermal stability of RPUFs was improved with the addition of alkanolamide polyol into the system. Thus, the RPUFs made from alkanolamide polyol are potential candidates to be used as insulation for refrigerators or freezers.

## 1. Introduction

Polyurethane (PU) is a versatile polymer that has been employed in a wide range of applications such as coatings, adhesives, foams, elastomers and others. With a proper selection of reactant, PU product ranges from high-performance elastomers to tough and rigid plastics can be easily fabricated [1]. Polyurethane foams (PUFs) are among the most important class of specialty PU. They can be divided into two major classes, which are flexible and rigid polyurethane foams [2]. Foams are also divided into categories depending on their pore morphology (open or closed) [3]. The RPUFs have attracted more and more interest over recent years because of their excellent thermal insulating properties, low apparent density and good resistance to various weather conditions [4,5]. PUFs are widely applied in multiple industries such as construction, cosmetics, bedding and automotive [6]. The ability to adhere to materials such as wood, steel, thermosetting resins and fibers, alongside resistance to oils, petroleum and other non-polar solvents are some other unique characteristics of RPUFs [7]. Therefore, due to their outstanding properties, RPUFs are used as insulating materials in refrigerators and freezers, and in the construction industry as well.

Basically, PU’s backbone structure consists of a soft segment of polyol and a hard segment of isocyanates. Polyols are compounds containing multiple hydroxyl functional groups. As one of the major feedstocks for PU production, most polyols used today in the PU industry are petroleum derived. Concerns about depletion and price increases of the world’s petroleum resources have led to increased research and industrial interests in developing bio-polyols from renewable resources as alternatives to conventional petroleum-based polyols [8]. In addition, a replacement of petrochemical components by low-cost natural oils allows a reduction of the carbon footprint. Thus, for economic and environmental reasons, the use of vegetable oils in polyol synthesis has been investigated by many researchers.

Bio-polyols can be obtained from different types of vegetable oils. The chemical structure of oils allows two types of modification in their molecular structures: modifications in ester bonds and/or in alkene groups [9]. A well-known method based on epoxidation of alkene groups can produce polyols with different chemical structures through epoxide ring-opening with components containing an active hydrogen atom [10,11]. This method is very often used given its industrial applicability and low cost. Other methods are transesterification with triethanolamine and transamidation with diethanolamine [11,12]. However, most vegetable oils are classified as the first generation of bio-based raw materials. This means that the synthesis of polyols based on edible oils is competing with the production of food. One of the main non-food applications of vegetable oils is biodiesel; for this application, vegetable oil is converted to fatty acid methyl ester (FAME), whereas methyl oleate (MO) is one type of FAME that is derived from transesterifications of vegetable oils and fats. Due to the large palm oil supply in Malaysia, this condition has driven Malaysia to develop biodiesel technology and production. According to Tuan Ismail et al., Malaysia has a capability to produce roughly about 10.2 million tons of biodiesel [13]. The low level of public support and rising nontariff barriers, which indirectly affect the demand for palm oil biodiesel, presented some difficulties for the Malaysian biodiesel industry. Therefore, the surplus supply and capacity of the biodiesel industry could be reduced by diversifying MO into other chemical compounds. On the other hand, the majority of bio-polyol obtained through chemical modification of vegetable oil is almost solid or semi-solid at room temperature, which is a major drawback for the production of rigid polyurethane foams [14]. The manufacture of RPUFs requires polyol to be in liquid form so it can be easily blended with isocyanates. Recently, the successful synthesis of RPUFs from vegetable-oil-based polyol derived from palm oil, rapeseed oil, castor oil and mustard oil has been reported [9,15,16,17]. Moreover, partial replacement of petroleum-based polyol is an alternative approach for the production of rigid polyurethane foams [7,15,18]. Kuranska et al. [19] prepared RPUFs by partially replacing up to 30% of the petroleum-based polyol with rapeseed oil bio-polyol; a reduction in the reactivity and the compressive strength of the mixtures obtained was observed. The RPUFs had a lower apparent density compared with the reference foams. However, the introduction of bio-polyol into RPUF formulation did not show any influence on the thermal conductivity coefficient compared with the reference foams, which was around 0.023 W/m.k. Adnan et al. [7] reported that as palm-oil-based bio-polyol was increased up to 50%, the thermal conductivity increased from 0.023 to 0.034 W/m.K. with an improvement in compressive strength. Marcovich et al. [20] found that by blending up to 70% of palm-oil-based bio-polyol with a petrochemical polyether polyol, the thermal conductivity of the RPUFs was higher and closed cell content lower in comparison with the reference foams—even the bio-foams presented a lower apparent density. 

To the best of the authors’ knowledge, there have been few works that reported on the production of RPUFs from palm oil, rapeseed oil and other vegetable oils [11,21,22]. However, no research has been reported on the production of RPUFs from bio-polyol synthesized from palm oil derivatives—namely, methyl oleate with hydroxyl value of 300–400 mg KOH/g, which fulfills the required hydroxyl value of polyol in making RPUFs. Additionally, no research has reported on the physical, mechanical and thermal properties of RPUFs made from methyl-oleate-based alkanolamide polyol. Therefore, this article reports on the synthesis of methyl-oleate-based alkanolamide polyol through a series of reactions, which are the epoxidation reaction, ring-opening with glycerol and amidation reaction with a hydroxyl value higher than 300 mg KOH/g; subsequently, the physical, mechanical and thermal properties of RPUFs made with this polyol were characterized considering their potential as thermal insulating materials.

## 2. Materials and Methods

### 2.1. Materials

MOG-polyol was prepared based on previous work by Kamairudin et al. [23]. Diethanolamine 99.5%, sodium chloride 90%, sodium hydrogen carbonate 90%, chloroform 99% and sodium methoxide 33% were purchased from R&M Chemicals (Shah Alam, Malaysia); starch solutions 1% and Wijs reagent were purchased from Systerm (Shah Alam, Malaysia); Dabco DC193, Dabco^®^ 33LV (33% triethylenediamine in dipropylene glycol) and Niax^®^-A1 (Bis (dimethylaminoethyl) ether in dipropylene glycol with equivalent weight = 233.70) were purchased from Sigma-Aldrich (Hamburg, Germany); Desmodur 44V20L (NCO% = 31.5, Equivalent weight = 133.33) was purchased from Bayer (Shah Alam, Malaysia); YD6205 polyol was purchased from Arch Chemicals (Beijing, China). All chemicals and reagent used in the analyses were of analytical grade.

### 2.2. Preparation of MOAG-Polyol with Diethanolamine via Amidation Reaction

Amidation reaction was conducted according to the method described by Lin et al. [24] and Palanisamy et al. [25] with a few modifications. MOG-polyol (200 g, 1.804 mol), diethanolamine (228 g, 2.168 mol) and 0.25 wt% of sodium methoxide catalyst were weighed into a 1 L, three-neck, round-bottom flask equipped with a magnetic stirrer, a thermometer and distillation set up, and a silicone oil bath. The mixture was heated to 120 °C and stirred vigorously at 200 rpm for 3 h. The mixture was cooled to room temperature, dissolved in chloroform (250 mL) and washed with 1% sodium hydrogen carbonate followed by 10% sodium chloride until the mixture was free from sodium methoxide with a pH value of 7–8. The mixture was dried over anhydrous MgSO_4_. Chloroform was removed using a rotary evaporator, resulting a dark brown liquid labeled as MOAG-polyol with 86% yield. For this reaction, various mole ratios of MOG-polyol to diethanolamine, different amounts of sodium methoxide catalyst and different reaction times were studied to obtain the optimum reaction condition for producing a polyol with the highest hydroxyl value. 

#### FTIR and NMR Data of MOAG-Polyol

υ_max_/cm^−1^ 3358 (O-H), 2923, 2853 (CH_2_), 1740 (C=O), 1617 (C=ONH), 1465 (CH_2_), 1364 (CH_3_), 1047 (C-O), 723 (CH_2_); ^1^H NMR (600 MHz, CDCl_3_): δ_H_ = 3.76–3.74 (1H,m,CH_2_OCHOHCH_2_OH), 3.73–3.72 (2H,m,CH_2_OCHOHCH_2_OH), 3.67–3.60 (4H,m,O=CN(CH_2_CH_2_OH)_2_) 3.53–3.49 (1H,m,CH_2_CHOCHOH), 3.41–3.31 (4H,m,O=CN(CH_2_CH_2_OH)_2_), 3.26–3.17 (2H,m,OCH_2_CHOHCH_2_OH), 3.04–3.03 (1H,m,CHOCHOHCH_2_)_,_ 2.3–2.2 (2H,m,O=CCH_2_CH_2_), 1.50–1.44 (2H,m,O=CCH_2_CH_2_CH_2_), 1.44–1.34 (4H,m,CHOCH_2_CH_2_), 1.34–1.18 (20H,m,CH_2_CH_2_CH_2_), 0.79 (3H,t,CH_2_CH_3_); ^13^C NMR (150 MHz CDCl_3_): δ_C_ = 175.5 (CH_2_O=CN(CH_2_CH_2_OH)_2_), 84.2 (CHOCHOHCH_2_), 72.7 (OCH_2_CHOHCH_2_OH), 71.8 (CHOCH_2_CHOH), 71.1 (CH_2_OCHOHCH_2_OH), 63.5 (CH_2_CHOCHOH), 60.3 (O=CN(CH_2_CH_2_OH)_2_), 51.7 (O=CN(CH_2_CH_2_OH)_2_), 33.1 (CH_2_CH_2_C=O), 32.0 (CH_2_CH_2_CHOH), 31.0–28.8 (CH_2_CH_2_CH_2_), 25.2 (CH_2_CH_2_C=O), 22.6 (CH_2_CH_2_CH_3_), 14.1 (CH_2_CH_3_).

### 2.3. Characterization of Alkanolamide Polyol

#### 2.3.1. Wet Chemical Analysis

Wet chemical analyses were performed following the American Oil Chemists’ Society’s (AOCS’s) official methods. The iodine value (IV) was analyzed following the AOCS’s official method Cd 1d-92 in order to quantify the alkene group present in a sample; this was conducted on the MOG-polyol and MOAG-polyol. The acid value (AV) is defined as the amount of potassium hydroxide in milligrams needed to neutralize the free fatty acid in 1 g of sample. It was conducted according to the AOCS’s official method Te 2a-64. The hydroxyl value (OHV) was determined following the AOCS’s official method Cd 13-60 in order to quantify the hydroxyl group present in the sample. This analysis was performed on both bio-based polyols. The saponification value (SV) tests of MOG-polyol and MOAG-polyol were performed according to AOCS’s Official method Cd 3-25. SV analysis was used in order to quantify the ester group present in the sample. The moisture content of the polyol was determined using a Karl Fisher Titrator following ASTM D 4672-00.

#### 2.3.2. Fourier Transform Infrared Analysis

The vibrational spectroscopic studies were investigated using a Perkin-Elmer FTIR Spectrum100 (Walthman, MA, USA) with an attenuated total reflectance (ATR) accessory, equipped with a diamond crystal with an angle of incidence at 45°. FTIR was performed in the frequency range of 4000–650 cm^−1^ for 16 repeated scans with a resolution of 4 cm^−1^ in transmittance mode at 25 °C.

#### 2.3.3. Nuclear Magnetic Resonance Analysis

Nuclear magnetic resonance spectroscopy (^1^H NMR) and (^13^C NMR) was conducted using JOEL JNM-ECZ600R (Peabody, St. Louis, MO, USA) at 600 MHz and 150 MHz, respectively, at 27 °C. Deuterated chloroform was used as a solvent to dissolve the sample with approximately 10% *w*/*w* of sample. The ^1^H and ^13^C chemical shifts were recorded in ppm and referenced to residue chloroform peak at 7.26 ppm.

#### 2.3.4. Molecular Weight Determination

The molecular weight of the samples was determined using Gel Permeation Chromatography (GPC) on a Varian PL-GPC 50 Plus (Polymer Laboratories Ltd., Church Stretton, Shropshire, UK) equipped with a differential refractive index (DRI)/viscometer and an autosampler. A set of four Phenogel columns (5 μm particle size and porosities of 50, 100, 1000 and 10,000 Å) from Phenomenex (Torrance, CA, USA), covering an MW range of 10^2^–10^6^ Da, was used for separation. The sample was dissolved in THF with a concentration of 2 mg/mL. THF was used as eluent at a flow rate of 1 mL/min. Molecular weight was measured based on the universal calibration curve created using polystyrene standards with a range from 162 to 1 × 10^5^ Da. Cirrus GPC/SEC software was used to analyze the data. GPC was utilized to determine the molecular weight distribution and average molecular weight of sample. 

#### 2.3.5. Viscosity Analysis

Viscosity was measured using a Brookfield Digital Rheometer, Model DV-111+ (Brookfield Engineering, Middleborough, MA, USA) at 25.0 ± 1 °C, equipped with a Brookfield TC-500 water bath according to ASTM D4878-03 standard practice. The viscometer was calibrated with 5 cP silicone oil prior to analysis. Approximately, about 1 mL of sample was put on the sample platform for testing. A flat plate spindle (PP41) was moved on the sample in rotation mode with a gap of 1 mm. The viscosity was measured using plate–plate geometry mode and analyzed using Rheo Min Software 2.0. The viscosity value at the shear rate with maximum torque (%) was selected as it indicated the minimum measurement error.

### 2.4. Preparation of Rigid PU Foam

#### 2.4.1. Formulation of Rigid PU Foam Made Using Alkanolamide Polyol from MOAG-Polyol

Rigid polyurethane foams (RPUFs) were prepared using a one-shot approach with a typical base formulation. The synthesized alkanolamide polyol (MOAG-polyol) was blended with a polyol (YD6205), which is a sorbitol-initiated polyether polyol derived from petrochemicals (OHV = 360 mg KOH/g, viscosity at 25 °C = 2140 mPa∙s, molecular weight = 880 Da and equivalent weight = 155.83) in several ratios. The formulations of RPUFs are listed in Table 1. The described RPUF formulations were designed to obtain low-density foams with apparent densities of about 27–35 kg/m^3^. The blended polyols, amine catalysts (Niax-A1 and Dabco 33LV) and blowing agent (water and Dabco DC193) were combined in a plastic cup using a mechanical stirrer with a 3″ diameter mixing blade at a high shear rate for a minute or until the mixture became creamy. This premix was named component A. This step was adapted from a method described by Arniza et al. [15]. Component B was a technical diisocyanate, Desmodur 44V20L. The correct amount of polymeric methylene dipenylmethane diisocyanate (Desmodur 44V20L with NCO% = 31.5; EW = 133.33) was then added and the mixture was vigorously agitated for 7 s. The amount of isocyanate used relative to the theoretical equivalent amount is known as the isocyanate index. To guarantee a full reaction between the isocyanate and polyol, an excessive amount of isocyanate was used in this study. The isocyanate index used for this study was 1.1. The mixture was then swiftly poured into an open mold made of a plastic cup with size 9 cm × 9 cm × 9 cm, and data on the foaming process, including cream time, gel time, rise time and tack-free time, were noted. The foam demolded from the plastic mold after 10 min. The prepared foam was aged for 7 days before it was cut into the necessary test specimens according to specifications.

#### 2.4.2. Characterization of Rigid PU Foam

##### Fourier Transform Infrared Analysis (FTIR)

The vibrational spectroscopic studies of the alkanolamide foams were investigated using a Perkin-Elmer FTIR Spectrum100 with an attenuated total reflectance (ATR) accessory; the procedure was explained earlier in Section 2.3.2.

##### Foaming Process

The foaming reactivity was recorded using an electronic stopwatch to determine the characteristics of the processing time (cream time, free rise time, string gel time and tack-free time) in accordance with ASTM D7487. The cream time was recorded from the start of mixing components A and B until fine bubbles appeared; the free rise time was noted from the start of mixing components A and B until the foam stopped expanding; the gel time was the time from the start of mixing components A and B until long strings of tacky materials could be pulled away from the foam surface by the tongue depressor; and tack-free time was indicated from start of mixing components A and B until the foam surface could be touched by a tongue depressor without sticking. 

##### Apparent Density

The density of prepared rigid polyurethane foams (RPUFs) was determined based on the samples’ mass and volume according to ASTM 1622-03. The samples (50 mm × 50 mm × 25 mm) were weighed and their volume recorded to calculate the density in kilograms per cubic meter. Three specimens were tested and the average value was reported.

##### Compressive Strength

A Hounsfield S-Series Machine (Surrey, UK) was used to test the compressive strength and strain of the foams at 10% deformation with in-house Horizon software. The sample of foams with size of 50 mm × 50 mm × 25 mm (triplicate samples) was compressed between two flat plates at a rate of 10 mm/min with a maximum load-cell capacity of 1 kN. The test was carried out using a modified version of ASTM D 1621-00.

##### Dimensional Stability

The dimensional stability measurements of the foams were determined according to ASTM 2126-99. The samples were cut to dimensions of 50 mm × 50 mm × 25 mm. A vernier caliper was used to measure the length, width and thickness of the samples. The samples were kept in the controlled temperature chamber at 70 °C and 30 °C for 14 days. Three specimens were tested for each sample and the average values were reported.

##### Water Absorption

The water absorption test determines the water absorption of foams by measuring the changes in buoyant force caused by immersion in water for 96 h. This test technique was carried out in accordance with ASTM D 2842-01. The length, height and width of samples were recorded before they were immersed into a beaker with distilled water; samples were removed from the beaker after 96 h. The length, height and width of the sample after immersion were recorded too. A sample size of 50 mm × 50 mm × 25 mm was used. Three specimens were tested for each sample and mean values were reported. The water absorption index was calculated based on the difference of volume in percentage.

##### Closed Cell Content

The volume of the sample block was determined using a Micrometrics Accu Pyc 1330 Pycnometer (Norcross, GA, USA) based on the change of pressure of a calibrated volume of nitrogen. Two sample cubes (25 mm × 25 mm × 25 mm) were put into the pycnometer cylinder to measure the gas displacement volume (V_p1_). The second volume (V_p2_) was then calculated by cutting each cube into four smaller cubes and returning them to the cylinder. Volume of the open cell (V_oc_) was calculated using the following Equation (1):

V_oc_ = 31.25 − 2V_p1_ + V_p2_ (1)

and the percentage of the open-cell content (V_oc_) was calculated as Equation (2):

(2) $${\%V}_{\mathrm{oc}}=\left(\frac{V_{\mathrm{oc}}}{31.25}\right)\times 100$$

in which 31.25 was the geometric volume of the samples. Therefore, the percentage of the closed cell content (V_co_) is as in Equation (3):

%V_co_ = 100 − V_oc_ (3)

##### Morphology Analysis

The morphology of RPUFs was analyzed using a JOEL JSM-6360 LA (Tokyo, Japan) scanning electron microscope. A thin piece of RPUF was carefully sliced with a sharp blade and stuck to aluminum stubs. Then, the samples were sputter-coated with a total of 15 nm of Au/Pd and observed under the microscope employing an accelerating voltage of 10 kV. The magnification used to observe the RPUFs was 50 nm. The statistical analysis of the six distributions of the RPUF cell was calculated on the basis of SEM images using ImageJ Software (Java 1.8.0_112, Media Cybernatics Inc., Rockville, MD, USA).

##### Thermal Conductivity

A heat flow meter HFM 436 Lambda (Netztech, Germany) was used to measure thermal conductivity (*k*) value of the foams in accordance with ASTM C 518-02. Samples were placed vertically between two brass plates. Heat was given from the top and directed downwards to prevent any convection within the sample. Measurements were collected after the samples had reached equilibrium, which took about 1–2 h. Samples with dimensions of 300 mm × 300 mm × 25 mm were used.

##### Thermal Gravimetric Analysis

The thermal stability analysis of the RPUFs was studied using a TG Analyzer, Perkin-Elmer TGA7 (Hamburg, Germany). TGA provides quantitative measurements of mass change in material associated with transition and thermal degradation as a function of temperature or time. About 6 mg of RPUF was heated from 25 °C to 800 °C at a constant heating rate of 10 °C/min under a nitrogen atmosphere (flow rate: 50 mL/min). Weight loss of the sample was plotted as a function of temperature.

## 3. Results and Discussion

### 3.1. Production of Alkanolamide Polyol (MOAG-Polyol) via Amidation Reaction

#### 3.1.1. Reaction Mechanism

**Figure 1:**
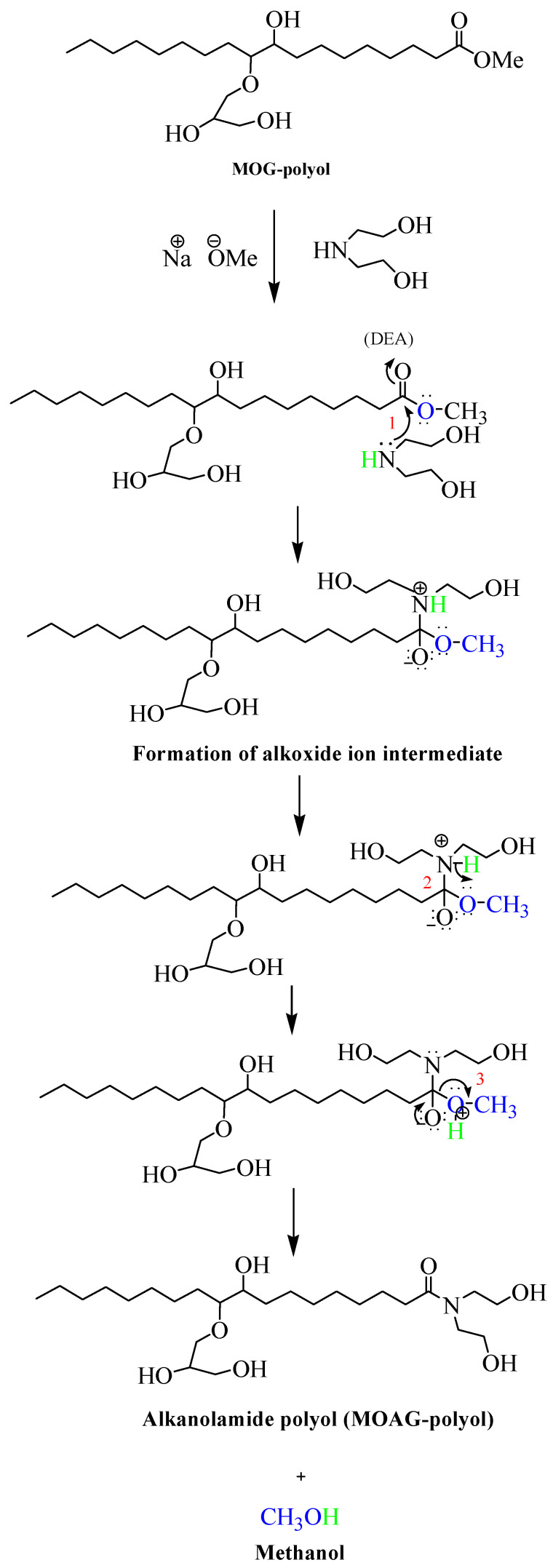
Reaction mechanism of amidation reaction of MOG-polyol.

The mechanism of amidation reaction of MOG-polyol with DEA in the presence of NaOMe as the catalyst is illustrated in Figure 1. For the first step, the electron lone pair from the nitrogen atom in DEA, was the nucleophile that attacked the carbon atom in the carbonyl group of MOG-polyol. Therefore, an alkoxide intermediate was formed, followed by deprotonation of the N-H bond by methoxide ion and the formation of methanol, as in Step 2. Finally, elimination of the alkoxide ion took place, leading (Step 3) to elimination of the methanol and formation of alkanolamide polyol, namely, MOAG-polyol.

#### 3.1.2. Optimization of Reaction Parameters of Amidation Reaction (MOAG-Polyol)

##### Effect of Molar Ratio Reactant: DEA on the Properties of Alkanolamide Polyol (MOAG-Polyol)

For this section, MOG-polyol was used as the starting material (reactant) for the amidation reaction with diethanolamine (DEA), which was conducted at 120 °C, and catalyzed by sodium methoxide (NaOMe). The first reaction parameter studied was the molar ratio of MOG-polyol: diethanolamine, which was used in the amidation reaction, and its effect on the properties of alkanolamide polyol, named MOAG-polyol, as shown in Group 1 of Table 2. It is interesting to find that a molar ratio of 1:2 of MOG-polyol to DEA with catalyst loading 0.25% over 3 h reaction time gave the highest hydroxyl value with the lowest polydispersity index. However, when the molar ratio was increased to 1:3 MOG-polyol to DEA, the OHV and molecular weight of MOAG-polyol were reduced slightly. This is attributed to the addition of excessive diethanolamine that causes intermolecular forces, such as hydrogen bonding and dipole attraction between the unreacted DEA with the hydroxyl group of the polyol compound, resulting in lower reactivity of the reaction mixture [26]. In addition, the excess amount of DEA diluted the catalyst concentration, and this might have lowered its strength to catalyze the reaction, which generated the lower OHV and molecular weight. 

##### Effect of Catalyst Dosage on the Properties of Alkanolamide Polyol (MOAG-Polyol)

Referring to Table 2 (Group 2), when the amidation reaction was conducted at 1:2 molar ratio of MOG-polyol to DEA and with catalyst dosage 0.15% (Entry 4), the resulting MOAG-polyol exhibited a hydroxyl value of 285 mg KOH/g. In comparison, when the reaction was repeated at a higher catalyst dosage (0.25%) (Entry 5), OHV become higher. However, it is interesting to note that in the reaction conducted with higher catalyst dosage to 0.5% (Entry 6), the resultant polyol showed a similar OHV to the polyol from Entry 5. This indicated that 0.25% of the catalyst was sufficient for the amidation reaction to achieve a desirable OHV.

##### Effect of Reaction Time on the Properties of Alkanolamide Polyol (MOAG-Polyol)

The amidation reaction of MOG-polyol was repeated at different reaction times to evaluate the effect of reaction time on the properties of alkanolamide polyol, as shown in Table 2 (Group 3). A longer reaction time generated a polyol with slightly higher molecular weight and PDI. The increment of PDI indicates the occurrence of a side reaction, which produced a polyol with higher chain length variability 

#### 3.1.3. Physicochemical Analysis and Molecular Weight Determination

Table 3 illustrates the physicochemical properties and molecular weight distribution for alkanolamide polyol (MOAG-polyol). The synthesized alkanolamide polyol exhibited a low acid value, which will not affect the polyurethane foaming procedure [27]. Upon amidation reaction, the hydroxyl value of the MOAG-polyol was increased, whereby the OH groups were contributed by diethanolamine. The increase in OHV can be seen in the FTIR spectra of MOAG-polyol, which showed a hydroxyl band at 3358 cm^−1^ that was broader compared with MOG-polyol. The reduction in saponification value indicated that the ester linkage was converted to amide group. The saponification value was significantly reduced but still considerably high, which suggested that not all ester linkages were converted to amide group. Therefore, it can be concluded that this amidation reaction only occurred partially. The high viscosity of the MOAG-polyol is explained by the hydrogen bonding of the polar amide group present in the polyol chemical structure. Based on the MW properties result as stated in Table 3, the synthesized MOAG-polyol could be classified as low MW bio-polyol (below 1000 Da), which is suitable for rigid PU foam [28], meaning that the alkanolamide polyol with low molecular weight was successfully synthesized. The alkanolamide polyol (MOAG-polyol) was in liquid form at room temperature and did not crystallize over time, which is a major difference compared with the bio-polyols obtained from palm oil [14,15].

#### 3.1.4. Structure Analysis by FTIR

**Figure 2:**
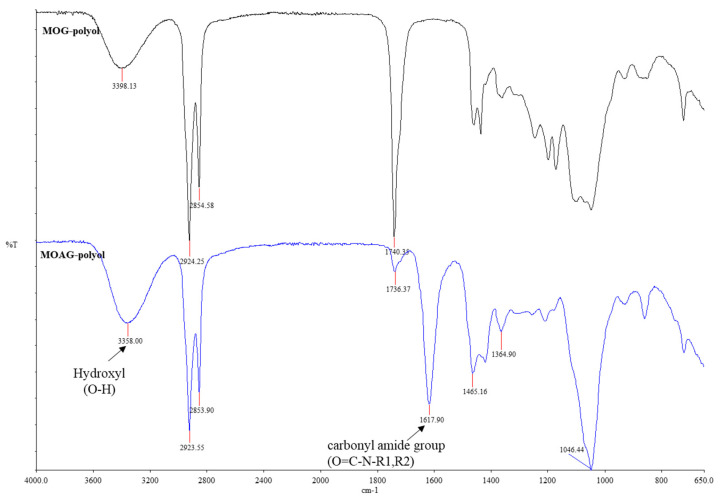
FTIR spectra of MOG-polyol and MOAG-polyol.

The FTIR spectra of MOG-polyol and MOAG-polyol are illustrated in Figure 2. As shown in Figure 2, after amidation reaction, the intensity of the peak at 3358 cm^−1^ (MOAG-polyol) was increased due to the increase in OH groups, which was due to the contribution of diethanolamine (DEA). These results also correlated with the OHV analysis, which recorded an increase in OHV after amidation with DEA. The peak at 2923 cm^−1^ and 2853 cm^−1^ were indicated as CH_2_ symmetric and asymmetric stretching [29]. There is a new peak at 1617 cm^−1^ in the MOAG-polyol spectra, which indicates the presence of C=O stretching of the carbonyl amide group that was generated from the amidation reaction of fatty ester. The C-N stretching of tertiary amide group and C-O stretching of ether group were observed at 1100–1020 cm^−1^. Both peaks overlap and could not be separated without further peak deconvolution. Similar observation of amide group was also reported in literatures [12,24,29]. In addition, the low peak of C=O stretching carbonyl ester at 1736 cm^−1^ supported the low ester group content as well as the decreased saponification value of MOAG-polyol. The slight shift of C=O stretching, from a peak of 1740 cm^−1^ to 1736 cm^−1^ is explained by the change of methyl oleate fatty acid structure to DEA fatty acid ester [30]. Peaks at 1465 cm^−1^ and 1364 cm^−1^ indicate the CH_2_ bending and CH_3_ bending, respectively; meanwhile, 1046 cm^−1^ represents the C-O-C ether groups in the polyol [13,29]. 

#### 3.1.5. Structure Analysis by NMR

**Figure 3:**
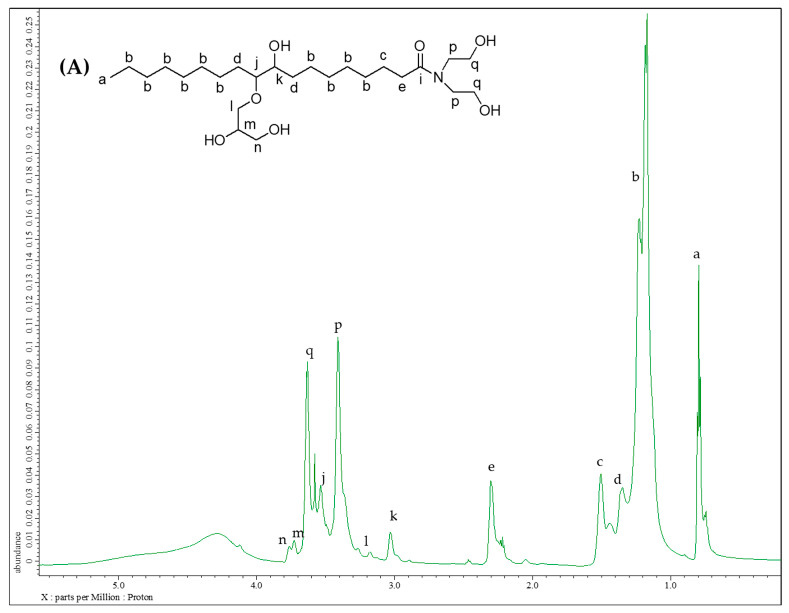

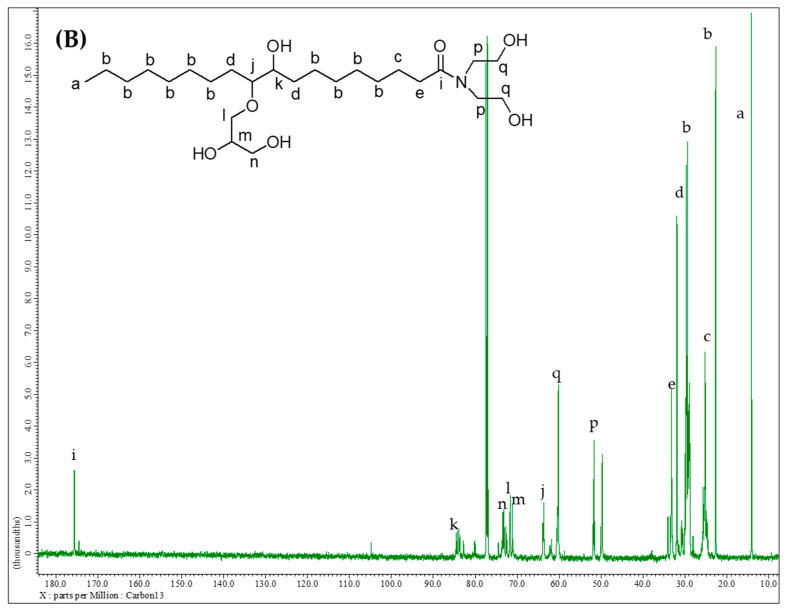
(**A**) ^1^H NMR spectra and (**B**) ^13^C NMR spectra of MOAG-polyol.

Figure 3A and Figure 3B revealed the ^1^H NMR and ^13^C NMR of MOAG-polyol, respectively. As shown in Figure 3A, new proton signals at 3.67–3.60 ppm (peak p) and 3.42–3.31 ppm (peak q) indicated that new OH groups were generated after the amidation reaction with DEA [31]. Moreover, as conformation, at signal 60.3 ppm (peak q) and 51.7 ppm (peak p) were observed in ^13^C NMR spectra, which indicated the presence of dibutylamide moiety in the prepared MOAG-polyol.

### 3.2. Production of Rigid Polyurethane Foams from MOAG-Polyol

#### 3.2.1. Foaming Process

The foaming process is a very crucial stage in the preparation of cellular materials. At this stage, the cellular structure is formed and has an influence on the physical–mechanical properties, such as thermal conductivity [17]. The foaming process of RPUFs was monitored by measuring appropriate processing stages such as cream time, rise time, gel time and tack-free time to investigate the reactivity of the developed RPUFs. Cream time is an indication of the beginning of a foaming process (generation of carbon dioxide gas), characterized by a visual observation of the start of foam expansion [32]. Therefore, due to the use of bio-polyols with different physicochemical properties, it was necessary to analyze the influence of these components on the foaming process. During the foaming process, the reaction mixture increases in volume several dozen times due to the formation of cellular structure [19]. Change in cream time, gel time and tack-free time indicates the reactivity of the polyol system.

The evaluation was carried out using a formulation of RPUFs with the same amount of additives and catalysts but varying in the content of the alkanolamide polyol, MOAG-polyol. The results are tabulated in Table 4. With the introduction of alkanolamide polyols, MOAG-polyol causes the foaming and gelation reactions to proceed faster than those of the reference RPUF, resulting in higher reactivity of RPUFs from bio-polyol compared with the reference RPUF. This is clearly observed as, with the increase in alkanolamide polyol, cream time and gel time decreased from 20 to 15 s and 58 to 40 s, respectively. Interestingly, this observation contrasts with previously reported data concerning the introduction of a rapeseed-oil-based polyol [5], which led to a decrease in the RPUF system’s reactivity. The effect observed here might be related to the higher viscosity of the bio-polyol used. The effect of the higher viscosity of polyol is also reflected by a lower value of the free rise time of the RPUFs, as bio-polyol content increased during the foaming process [33]. In addition, Devi et al. [34] reported that palm-based polyol or castor oil reacted faster than petroleum-based polyol. This may be due to better compatibility between natural oil polyols with isocyanate in comparison with polyether polyol, as mentioned by Felipe et al. [35], who suggested that there is poor miscibility between petroleum-based polyol and isocyanate due to polarity and density. Kuranska et al. [19] observed that the reactivity of RPUFs also increased due to the remaining alkaline catalyst from the synthesis of bio-polyol. Therefore, in this study, MOAG-polyol may have a remaining alkaline catalyst (sodium methoxide) that increased the reactivity of RPUFs. 

**Figure 4:**
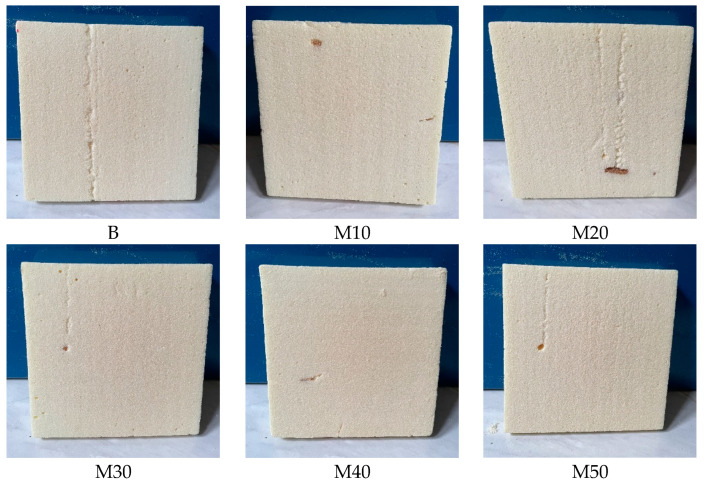
Physical appearance of RPUFs.

It is also reported that a higher OH value results in longer reaction time because with more OH groups per molecules, the molecular mobility and foaming efficiency are reduced [36]. It is clearly observed that the reference RPUF has a higher reaction time compared with the RPUFs containing alkanolamide polyol. It is interesting to note that Paruzel et al. [37] found that the faster foaming or gelling due to the addition of recycled polyol might yield RPUFs with smaller cell sizes. Figure 4 shows the series of RPUFs developed in this work.

#### 3.2.2. Structure Analysis by FTIR

**Figure 5:**
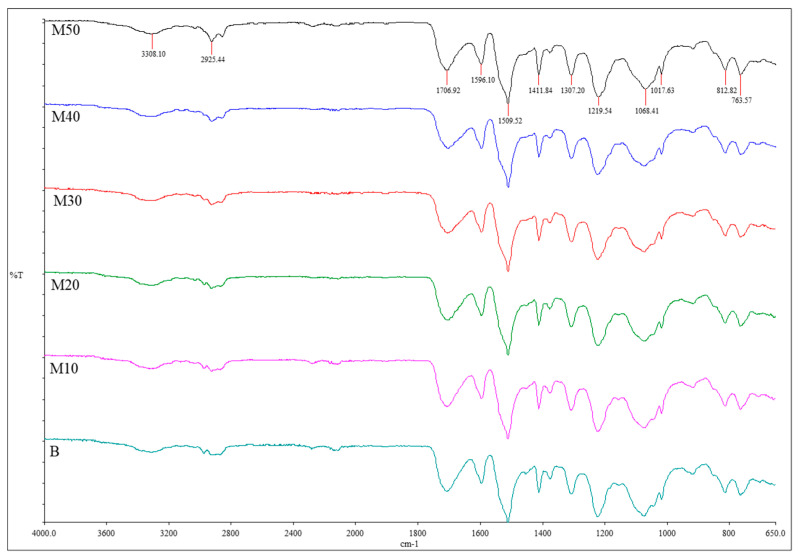
FTIR spectra of rigid polyurethane foams from MOAG-polyol.

FTIR spectra were used to identify the presence of functional groups for the benchmark and alkanolamide polyurethane foams, which confirm the correct course of the reactions. In general, the reaction reveals the fundamental group of the polyurethane polymer, which consists of urethane and urea linkages, uretoneimine ring structure and unreacted isocyanate [7]. Figure 5 depicts the full FTIR spectra of polyurethane foams made up from MOAG-polyol, and the major band observed in FTIR with the assignment to the chemical group is summarized in Table 5.

In general, the chemical structure of the manufactured foams is similar for all formulations from MOAG-polyol. The characteristic peaks indicating the formation of urethane linkages were observed. They are sharp and strong peaks of N-H stretching and bending at signals 3319–3308 cm^−1^ and 1510–1519 cm^−1^, respectively; a strong hydrogen-bonded urethane carbonyl (C=O) peak at 1708–1703 cm^−1^, which confirms that the urethane linkage is successfully formed [16]; another sharp and strong peak at 1224–1220 cm^−1^ attributed to the C-N stretching mode; and a peak at 1074–1068 cm^−1^ representing the C-O-C stretching mode [21,38]. All these peaks provide strong evidence for the formation of polyurethane [39]. Meanwhile, the characteristic peaks at signals 2929–2924 cm^−1^ and 2855–2852 cm^−1^ are attributed to the symmetric and asymmetric stretching vibration mode of the C-H bond in the CH_3_ and CH_2_ groups [40]. Interestingly, the peak in the range of 2269–2275 cm^−1^, which corresponded to the stretching vibration of N=C=O, nearly disappeared, which suggests that isocyanate groups reacted quantitatively with the hydroxyl group of polyols to form urethane bonds, as would be expected at an isocyanate index of 1.1 [7,41]. The signal at 1596–1594 cm^−1^ corresponds to the presence of aromatic rings in the foams while the signal at 1411 cm^−1^ indicates the presence of isocyanate trimerization products [40].

#### 3.2.3. Apparent Density

**Figure 6:**
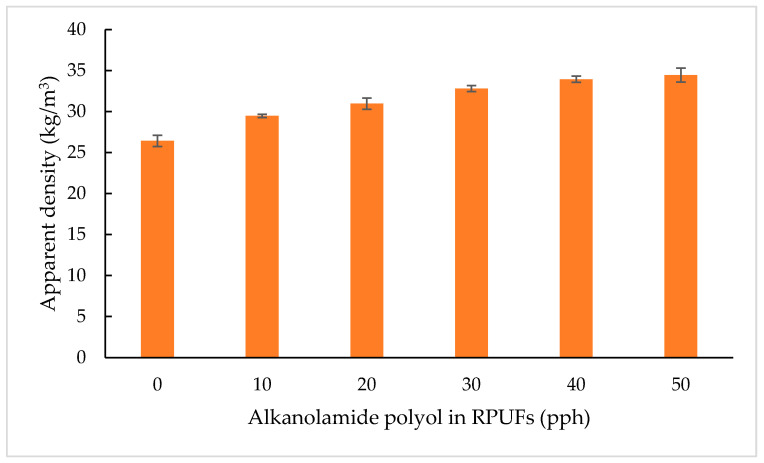
Apparent density of RPUFs.

Apparent density is a crucial factor that influences the thermal and mechanical properties of the rigid polyurethane foams (RPUFs) [6]. In order to study the influence of the amount of alkanolamide polyol (MOAG-polyol) on the properties of RPUFs, all foams were prepared by the same method and the content of the other reagents kept constant. Figure 6 shows the apparent densities of alkanolamide RPUFs with various MOAG-polyol content. The apparent densities of RPUFs increased relatively by 19.53%, from 28.83 to 34.46 kg/m^3^, as the content of MOAG-polyol was increased from 0 to 50%. This pattern was attributed due to the increasing viscosity of the polyol blends due to the increase in bio-polyol content [25]. The increase in polyol blend viscosities limited the expansion during the foam formation and also restricted bubble formation [16,42]. Similar results were reported by Polaczek and co-workers, where the highest viscosity of polyol blends in the production of open-cell PU foams from used cooking oil gave the highest apparent density [43]. As clearly stated in the previous section, the viscosity of MOAG-polyol is 11 times higher compared with the viscosity of petroleum-based polyol. Therefore, the apparent density of RPUFs is higher in comparison with the reference RPUF due to the lower viscosity of petroleum-based polyol.

Ryszkowska and co-workers observed a similar influence on the apparent density, such that the use of different bio-polyols based on rapeseed oil also increases the apparent density of foams based on them. They showed that the reason for this is that the use of high-viscosity bio-polyols leads to the formation of open cell structure and less-regular foams [39]. RPUFs with a density lower than 24 kg/m^3^ are not particularly stable dimensionally, and RPUFs with a density lower than 64.07 kg/m^3^ are categorized as having low apparent density. Therefore, the resulting RPUFs from MOAG-polyol can be classified as low-density foams. The use of materials with lower apparent density is advantageous from an economic point of view [25].

#### 3.2.4. Compressive Strength

Compressive strength is an important parameter of rigid polyurethane foams because they are most often used as thermal insulation materials that must withstand compressive loads under their operating conditions. Deformations can often be observed when using foams, especially when they are exposed to alternating high and low temperatures. This is due to the pressure of the gas filling the cells under the influence of temperature variation. The foam will not deform if its compressive strength exceeds 100 kPa, i.e., the pressure difference that can occur between atmospheric pressure and the pressure inside the foam cells (assuming complete condensation of the blowing agent) [17]. Generally, the compressive strength of RPUFs is influenced by several parameters, including foam apparent density and foam morphology (cell size and closed cell content) [27,44,45].

**Figure 7:**
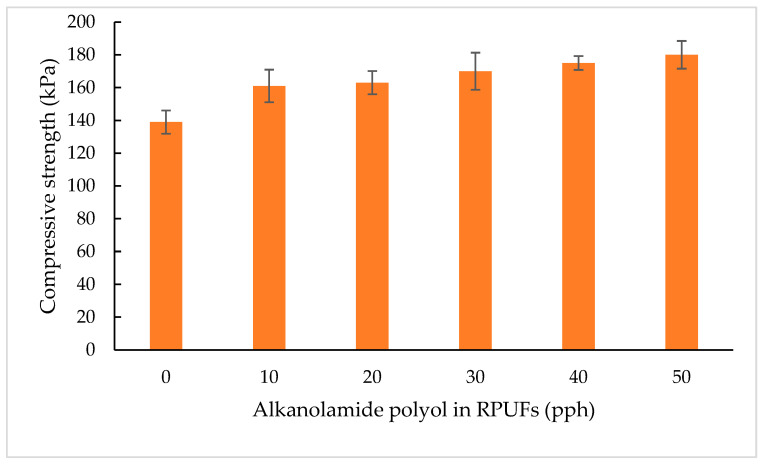
Compressive strength of RPUFs.

As presented in Figure 7, the compressive strength of RPUFs increased with higher content of the alkanolamide polyol. The compressive strength of the RPUFs with various amounts of alkanolamide polyol is in the range of 138–180 kPa. It worth noting that RPUFs that contained 50% of MOAG-polyol (M50) showed a strength improvement of 23% compared with the reference foam (B); this is due to the higher apparent density of RPUFs containing alkanolamide polyol in comparison with the RPUF containing 100% petrochemical-based polyol. Higher apparent density is attributed to the smaller cell size of RPUFs. As the cell size of RPUFs decreases, the cell wall thickness increases, which increases the compressive strength [46]. 

Hejna et al. [47] concluded that incorporation of bio-polyol, which was produced by polymerization of crude glycerol and further condensation with castor oil, led to an increase in foam density due to the reduction in the average cell size of RPUFs from 372 to 275 μm. Septevani et al. [21] also reported that compressive strength is closely related to the apparent density and cell size of the RPUFs. In their work, they concluded that with the decrease in apparent density, the compressive strength decreased as well due to the larger cell structure that caused a weaker cell wall. In addition, the higher viscosity of the polyol blended also leads to improvements in the compressive strength [16,39].

In a study reported by Narine et al. [44], the type of cells—namely, open or closed cells—also influenced the compressive strength. As stated in Table 6, the reference RPUF (B) has the highest closed cell content (48%) compared with the other RPUFs. It is known that the strength of the open cell structure of RPUFs is greater than that of the closed cell structure, which has had their wall ruptured and, due to the broken walls, are unable to contribute any reinforcement to support stress when the network is under load. Therefore, an addition of alkanolamide polyols to the RPUFs system increased the open cell structure, which led to an increase in the compressive strength. Nevertheless, all the RPUFs in this study are within the acceptable limits. According to Adnan et al. [7] and Ivdre et al. [45], the compressive stress for typical industrial RPUF insulating foam is between 100 and 250 kPa.

#### 3.2.5. Dimensional Stability

Rigid polyurethane foams, due to their ability to be molded to size, are particularly suitable for structural or foamed-in-place applications, where a complex shape is involved. The dimensional stability of rigid polyurethane foams is the most important property to be carefully considered, especially for low-density foams [19,48]. As an example, for thermal insulation materials, either a sandwich panel or household electric appliance, its dimensions must be maintained with time for functional and aesthetic reasons. Suitably selected thermal insulation directly affects the saving of energy costs and the correct operation of the technological installation [17].

Dimensional stability is the result of a balance between the pressure force and strength of a polymeric matrix [49]. In this study, the trends of foam dimensional and weight variations at 70 °C and −20 °C from alkanolamide polyol (MOAG-polyol) were recorded for up to 14 days, as shown in Table 7 After thermal treatment (70 °C), the weight of RPUFs decreased as ageing increased with a 0.77–1.42% range of weight changes. In contrast, the volume of the RPUFs increased after the thermal treatment as well as the increase in ageing but did not exceed a 2% change. Similar findings were also reported in the literature [7,48,50]. According to Lin et al. [48], and Zieleniewska et al. [10], the diffusion of air into and the carbon dioxide out of cells were accelerated at a higher temperature. Changes in the external temperature affect the pressure of the gas trapped in the cells of the foam, which generates pressure differences between the cell interior and the atmosphere and causes the foam to deform. According to appropriate industrial standards (BS4370: Part 1: 1988), the cold store panel should have less than a 3% change in weight and volume when tested at 70 °C [21,27]. Therefore, the dimensional changes of all the foams in this study are considered mild, stable and acceptable for insulating purposes. 

#### 3.2.6. Water Absorption

**Figure 8:**
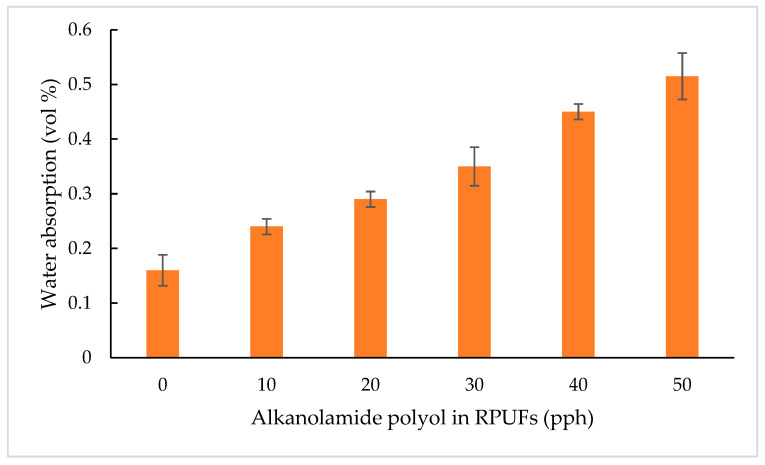
Water absorption of RPUFs.

Water absorption is another important parameter for RPUFs because the applications of RPUFs requires low water absorption, which is conducive to the drying of the products [6]. It is well-known from past studies that water absorption depends on the cell morphology of the RPUF’s either closed or open cell structure [51]. Therefore, water absorption of RPUFs with various percentages of alkanolamide polyol were investigated in this study. As illustrated in Figure 8, the addition of alkanolamide polyol influences the water absorption of RPUFs. As the amount of alkanolamide polyol in RPUFs was increased, the water absorption increased due to the higher content of the open cell structure of RPUFs. This observation can be associated with the high reactivity of the system and a tendency of weak cell walls in foamed materials to rupture due to the high pressure of the gas trapped in the foam cells. Therefore, the open cell structure was able to accommodate more water compared to the RPUFs with a well-developed closed cell structure [9]. In this work, the reference RPUF had the lowest water absorption in comparison with the RPUFs that contained alkanolamide polyol. This is due to having the highest closed cell structure compared with the other RPUFs. Thus, it is clear that the morphology of rigid PU foams was the main factor that affected the water absorption. A similar trend was also described in literature by Kairytė and Vėjelis [52], and Zieleniewska et al. [53]. 

#### 3.2.7. Cell Structure Morphology by SEM

**Figure 9:**
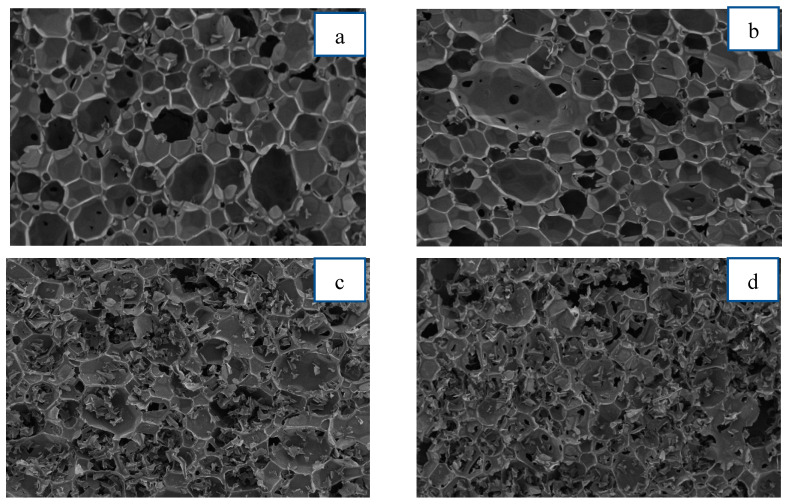

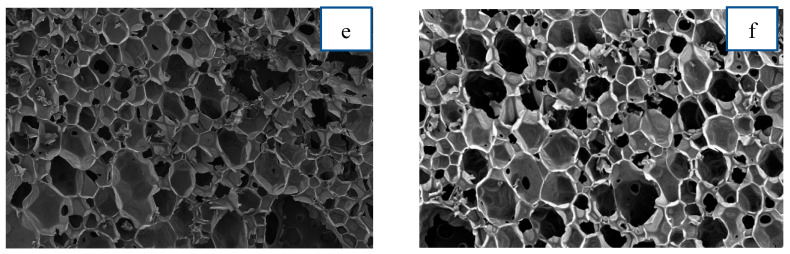
Micrographs of RPUFs from MOAG-polyol: (**a**) Ref, (**b**) M10, (**c**) M20, (**d**) M30, (**e**) M40 and (**f**) M50.

**Figure 10:**
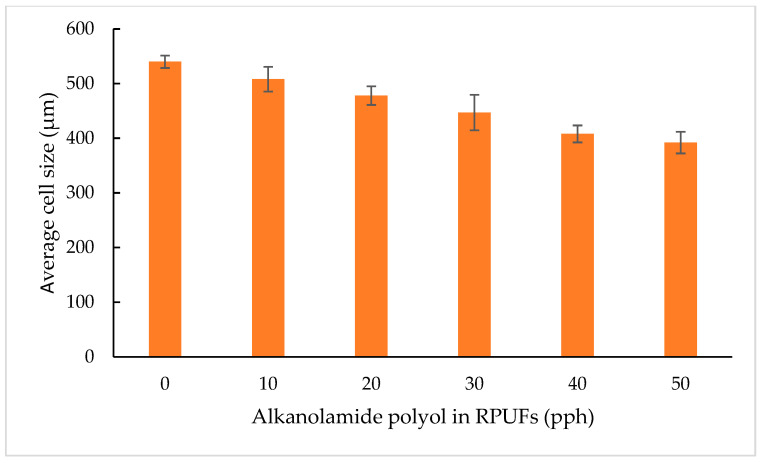
Average cell sizes of RPUFs.

The cell structure is a crucial parameter of the RPUFs, which influenced the properties of the obtained material directly or indirectly, as examples are thermal insulation and mechanical properties [17,18]. The influence of alkanolamide polyol at different percentages on the cellular structure of RPUFs was examined using SEM. The cell structures shown in Figure 9 are typical RPUFs prepared in this work from MOAG-polyol. The average cell sizes are listed in Figure 10. The average cell size was calculated from the size estimates of 100 cells identified in SEM. As observed, all RPUFs were composed in the shape of a polyhedral or sphere. Ruptures and debris can also be observed in all RPUFs, resulting from the cutting process during the sample preparation [42].

The reference RPUF (Figure 9a) showed the largest average cell size of 540 μm and the cell distribution is nearly uniform. As showed in Figure 9b–f, the average cell size of RPUFs decreased with the increase in MOAG-polyol content up to 50%. The cell distribution also became uneven or non-uniform as the percentage of MOAG-polyol increased. Hejna et al. [47] observed a similar dependence during the synthesis of RPU/PIR foams based on bio-polyol from castor oil and crude glycerol. They obtained a reduction in average cell size from 372 to 275 μm. Generally, the low viscosity of polyols leads to the formation of a larger cell size because it can be easily merged with adjacent cells due to the delayed cross-linking of the PU wall. On the other hand, at higher viscosity, the growth of the carbon dioxide bubble may be hindered by uneven and rapid crosslinking, resulting in heterogeneity in the morphology foam and smaller cell size, which is similar to this work [16,21]. As the content of alkanolamide polyols increased, the cell sizes of RPUFs decreased and the cell structure became non-uniform. It is worth noting that the viscosity of the polyol is critical to the preparation of the PU foam and cellular structure of the resultant foam. The cell size depends largely on the system viscosityat higher viscosity, it has an influence on restraining the expansion of the cells [27]. 

Moreover, the faster foaming and gelling time due to the addition of bio-polyol might also yield foam with smaller size due to faster polymerization [37]. It is interesting to note that with the same bio-polyol substitution, the tendency of cell size was just opposite with the apparent density; it is understandable that foam with smaller size generally has higher apparent density [4,27]. This is similar to the finding in this work.

#### 3.2.8. Thermal Conductivity and Closed Cell Content

Thermal conductivity (λ) is a vital attribute that governs thermal insulation applications of RPUFs, and it is essential to keep this value as low as possible [4,20]. The λ coefficient consists of four components: λ gas, λ radiation, λ solid and λ convection. The value of the λ coefficient of RPUFs is calculated as a sum of λ of the gas in the cell (λ gas), λ through the solid polymer (λ solid), the radiation heat transfer across the wall of the solid struts (λ radiation) and the convection of the gas within the cells (λ convection) [54]. In other words, the thermal conductivity of foam is affected by the conductivity of the PU phase (solid phase) as well as gas trapped (CO_2_ in this case because water was used as a blowing agent) within the closed cell content structure [4]; further, heat transport is affected by radiation between cells [11,21]. 

According to work reported by Septevani et al. [21], the thermal conductivity of RPUFs increases with larger cell size but decreases with higher closed cell content because open cells allow more convection and even allow air to enter the foam, which has a much higher conductivity value (0.0249 W/m.K) than that of CO_2_ (0.0153 W/m.K). As shown in Table 6, the reference RPUF (B) has the lowest thermal conductivity (0.034304 W/m.K) compared with the RPUFs made with alkanolamide polyol (0.035445–0.037045 W/m.K), even though it has the largest cell size. This is due to the high closed cell content of the reference RPUF. A similar observation was reported by Tu et al. [55] in their work. An addition of soybean oil-based polyol in the PUR foams also had an influence on the reduction of the closed cell content. The authors reported that a reduction in closed cell content percentage increased the thermal conductivity of the PUR foam by approximately 20%, which is similar to the findings of the current study. The observed higher open cell content in tandem with higher alkanolamide polyol content in RPUFs formulation can be caused by the reduction in crosslinking due to the lower hydroxyl value of alkanolamide polyol in comparison with petrochemical polyol.

Tan et al. [4] also reported that thermal conductivity is closely related to foam density and cell morphology. The low density of RPUFs is very beneficial for the performance of thermal insulation since the λ values for solid PU foam, CO_2_ and air are 0.222, 0.0153 and 0.0249 W/m.K, respectively [56]. In contrast, [20] reported that the addition of palm-oil-based polyol caused the apparent density of RPUFs to decrease; conversely, the thermal conductivity increased due to the reduction in closed cell content. For this current work, when the amount of alkanolamide polyol in RPUFs was increased, the apparent density also increased in tandem with the reduction in closed cell content, which resulted in increased thermal conductivity of the RPUFs. Ji et al. [27] also reported that an RPUF with high density had higher thermal conductivity because high density led to λ solid that was dominant over other factors although it possessed a small average cell size. 

Although a modification of RPUFs with alkanolamide polyol gave higher thermal conductivity of RPUFs with the highest value of 0.037045 W/m.K at 20 °C, it is worth noting that it still can be used as an insulating material as its thermal conductivity is less than 0.1 W/m.K [57].

In addition, the thermal conductivity depends strongly on the average temperature of the measurement. The higher the temperature, the higher the thermal conductivity. Generally, producers of RPUFs provide the value of thermal conductivity at the average temperature of 20 °C and the data for other conditions are rarely presented. Therefore, in this study, the thermal conductivity at different average measurement temperatures were provided because it is important from an industrial point of view [58]. Foam materials are used at different temperatures depending on the application, which affects the effectiveness of heat insulation.

#### 3.2.9. Thermal Degradation by TGA

**Figure 11:**
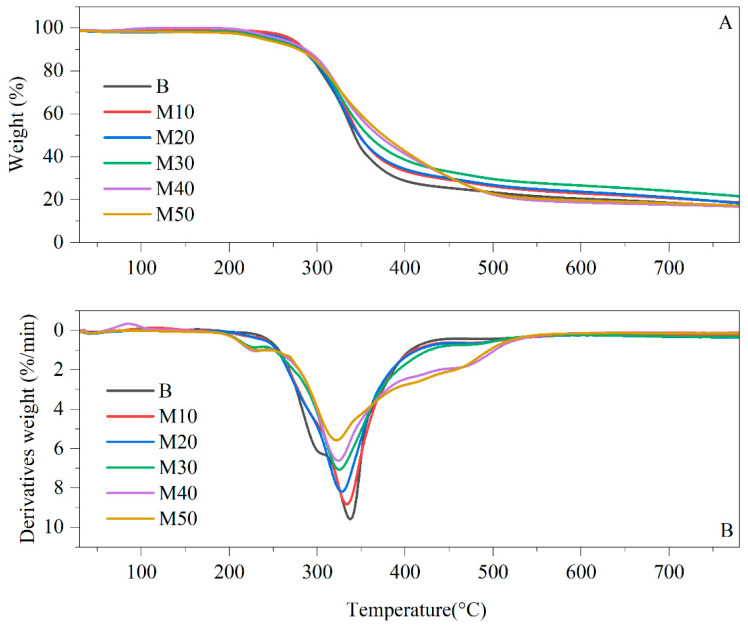
(**A**) TGA and (**B**) DTG thermograms of RPUFs from MOAG-polyol.

The thermal degradation analysis of material is important from scientific and technological points of view. Investigations of the thermal degradation of polymers are crucial in order to determine the proper conditions for manipulating and processing, and to obtain high-performance products that are stable and free of undesirable by-products [18]. Thermal resistance is related to the physical changes in rigid polyurethane foams (RPUFs) that occur under the influence of temperature. An increase in temperature leads to cracking of the weakest bonds in the RPUFs, causing polymer degradation and resulting in mass loss [9]. Therefore, a thermogravimetric study involving the analysis of the mass change signal (TG) as well as its derivatives (DTG) was employed to investigate the thermal stability of the developed RPUFs. In order to evaluate the behavior of RPUFs, TGA and DTG tests were conducted under a nitrogen atmosphere. The TGA and DTG curves of the RPUFs containing MOAG-polyol are presented in Figure 11. The summarized results of the thermogravimetric analysis of the materials are listed in Table 8.

For all the RPUF samples, the degradation started at approximately 200 °C and ended at 525 °C, as displayed in Figure 11. These findings are similar to those in the reported study by Kong et al. [59] on the thermal degradation of rigid PU foam from castor-oil-based polyol. It is worth noting that the thermal stabilities of PUs are dependent on the additives, reactants and conditions with which they were produced [59]. The weight lost for all RPUFs up to 230 °C corresponds to the moisture absorbed or easily volatilized products by the foams; thus, the mass loss in this temperature range is negligible [10]. The result of the analysis indicates that the introduction of the bio-polyol from MOAG-polyol caused a decrease in the temperature at 5% weight loss (T_5%_) of the foam thermal degradation in the range of 237–270 °C compared with the reference foam (273 °C), which is similar to studies reported by [60]. The lowest degradation temperature from bio-polyol was observed for the RPUFs containing 50% by weight of bio-polyol. The observed trend might relate to the higher thermal stability of the petrochemical polyol compared to bio-polyol, which means that bio-polyol is more susceptible to thermal degradation [60]. As shown in Table 8, the RPUFs from MOAG-polyol exhibited a significantly lower degradation temperature at 5% weight loss (from 237 to 270 °C) compared with the reference RPUF. 

The temperature at maximum degradation (T_max_) was displayed in DTG curves as well as in Table 8. The T_max_ of the reference RPUF (338 °C) has a higher temperature compared with RPUFs from bio-polyol (322–336 °C). The observed differences in thermal stability at T_max_ between RPUFs from petrochemicals and RPUFs from bio-polyol may be due to the structural differences between them. The RPUFs from petrochemicals contain only a primary hydroxyl group; meanwhile, RPUFs from bio-polyol contain a primary and secondary hydroxyl group. It is known that PU derived from the secondary hydroxyl group is less stable than those incorporating the primary hydroxyl group [59].

**Figure 12:**
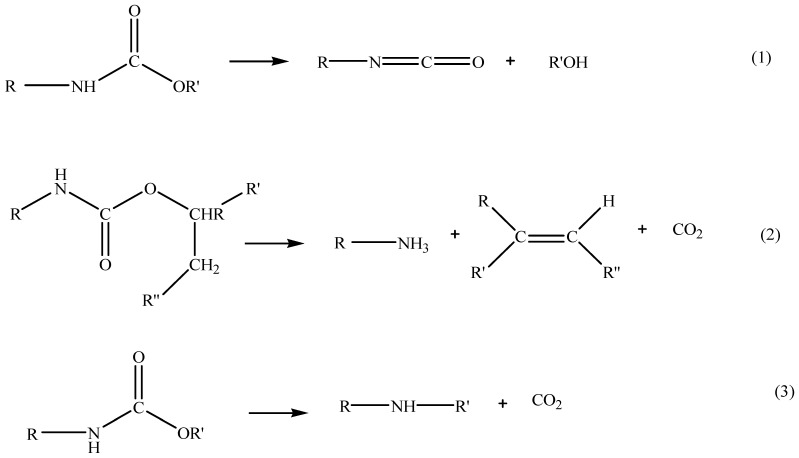
Basic reactions for thermal degradation of polyurethane.

According to the previous work data in Table 8, the first thermal degradation step that corresponded with temperature at 10% weight loss (T_10%_), which was from 278 to 285 °C, was related to the degradation of the rigid PU segments as well as degradation of the urethane linkage [42]. Rupture of hard segments takes place at a relatively low temperature, and great amounts of isocyanates are formed in the decomposition residues at the early stage [38,61]. The urethane linkage degradation involves three competing mechanisms: (1) the degradation of urethane to isocyanate and alcohol, (2) the formation of the primary amine and carbon dioxide and (3) the formation of the secondary amine and carbon dioxide, as shown Figure 12 [61]. 

The second thermal degradation of RPUFs is at the temperature of 50% of weight loss (T_50%_) in the range of 342–375 °C, which is related to the decomposition of the petrochemical polyol and bio-polyol contained in the flexible phase [62]. It is clearly observed that, at T_50%_, the decomposition of RPUFs shifts to a higher temperature as the amount of alkanolamide increases, as stated in Table 8.

The final residue of RPUFs at 780 °C clearly indicated that the RPUFs containing bio-polyol show high residue in comparison with the reference foam. It suggests that the introduction of bio-polyol (alkanolamide polyol) improves the overall thermal stability of RPUFs. Therefore, alkanolamide polyol was suitable for the preparation of rigid polyurethane foam with a higher decomposition temperature [22,63].

## 4. Conclusions

The rigid polyurethane foams (RPUFs) were successfully prepared and characterized from bio-polyols (alkanolamide polyol), namely, MOAG-polyol. The replacement of a virgin petrochemical-based polyol up to 50 wt% by the alkanolamide polyol to the polyurethane system increased their reactivity due to the higher viscosity of this bio-polyol. The low density of RPUFs with apparent density of 26–35 kg/m^3^ was obtained in this work. The compressive strength of the prepared RPUFs also increased with the increase in alkanolamide polyol due to the subsequent increase in the open cell structure of RPUFs. The modified RPUFs presented excellent dimensional stability and water absorption, which is less than 3% and 1% of the weight changes, respectively. The thermal conductivity of modified RPUFs with alkanolamide polyol was slightly higher than that of the reference foam due to the lower closed cell content as well as the formation of a less-regular cell structure. The micrographs show that the average cell size decreased with the increase in alkanolamide polyol due to the higher viscosity. The introduction of alkanolamide polyol improved the thermal stability of RPUFs. The thermal degradation at temperature of 50% weight loss (T_50%_) increased from 342 to 375 °C and increased the residual char after a thermogravimetric measurement. Therefore, the results presented in this work indicate high application potential of bio-polyol based on palm oil derivatives for the production of RPUFs dedicated for thermal insulation. 

## Figures and Tables

**Table 1:** Formulation of RPUFs containing MOAG-polyol.

Formulation Designation	B	M10	M20	M30	M40	M50
Ratio (pph) ^a^						
YD6205	100	90	80	70	60	50
MOAG	-	10	20	30	40	50
Water	4.5	4.5	4.5	4.5	4.5	4.5
Dabco DC193	2.0	2.0	2.0	2.0	2.0	2.0
Dabco 33LV	1.8	1.8	1.8	1.8	1.8	1.8
Niax A1	0.1	0.1	0.1	0.1	0.1	0.1
Isocyanate system						
Desmodur 44V20L	175.98	175.56	175.15	174.73	174.32	173.91
Isocyanate index ^b^	1.1	1.1	1.1	1.1	1.1	1.1

^a^ The ratio of all components are expressed in parts per hundred of polyol. ^b^ Isocyanate index is defined as the ratio of the equivalent amount of isocyanate used relative to the theoretical equivalent amount times 100.

**Table 2:** Properties of alkanolamide polyol.

Entry	Molar Ratio	Catalyst	Time	OHV	GPC		
	Reactant: DEA				M_w_	M_n_	PDI
Group 1							
1	1:1	0.25	3	279	570	509	1.12
2	1:2	0.25	3	313	664	600	1.11
3	1:3	0.25	3	300	575	517	1.11
Group 2							
4	1:2	0.15	3	285	600	547	1.10
5	1:2	0.25	3	313	664	600	1.11
6	1:2	0.50	3	292	623	525	1.20
Group 3							
7	1:2	0.25	2	270	571	527	1.10
8	1:2	0.25	3	313	664	600	1.11
9	1:2	0.25	6	274	701	619	1.13

Note: catalyst used is NaOMe (%). Time—reaction time (h); OHV—hydroxyl value of polyols (mg KOH/g); GPC—gel permeation chromatography; M_w_—molecular weight (Da); M_n_—number average molecular weight (Da); PDI—polydispersity index (M_w_/M_n_).

**Table 3:** Physicochemical properties and molecular weight determination of MOG-polyol and MOAG-polyol.

Parameters	MOG-Polyol	MOAG-Polyol
IV (g I_2_ 100/g)	5.38	6.76
AV (mg KOH/g)	1.18	2.33
OHV (mg KOH/g)	306	313
SV (mg KOH/g)	140	106
Viscosity at 25 °C (mPa∙s)	513	22,800
Moisture content (wt%)	0.10	0.84
MW (Da)	488	664
Average number, M_n_ (Da)	428	600
% Oligomer	20	17
PDI = MW/M_n_	1.14	1.11
Physical appearance	Liquid	Liquid

**Table 4:** Processing times of RPUFs.

Designations	Cream Time (s)	Gel Time (s)	Free Rise Time (s)	Tack-Free Time (s)
B	20	58	115	153
M10	17	48	99	128
M20	17	45	90	114
M30	16	42	84	93
M40	16	42	76	76
M50	15	40	68	68

**Table 5:** Band assignment for rigid polyurethane foams from MOAG-polyol.

100 PP/ 0 BP	90 PP/ 10 BP	80 PP/ 20 BP	70 PP/ 30 BP	60 PP/ 40 BP	50 PP/ 50 BP	
Wavenumbers (cm^−1^)						bond vibration
3314	3319	3314	3317	3318	3308	N-H stretching
2923	2927	2925	2926	2924	2925	C-H asymmetric
2853	2852	2854	2855	2854	2854	C-H symmetric
1708	1706	1704	1704	1703	1706	C=O stretching
1595	1595	1595	1595	1595	1596	C=C (aromatic, stretching)
1510	1510	1509	1509	1509	1509	N-H bending
1411	1411	1411	1411	1411	1411	PIR (deformation)
1222	1221	1221	1222	1222	1222	C-N (stretching)
1074	1073	1071	1073	1072	1068	C-O-C (stretching)
764	764	764	763	763	763	C-H (deformation)

Note: PP indicates petroleum-based polyol; BP indicates bio-based polyol.

**Table 6:** Thermal conductivity coefficient and closed cell content of RPUFs.

Designations	Thermal Conductivity Coefficient (W/m.K)			Closed Cell Content (%)
	20 °C	50 °C	70 °C	
B	0.034304	0.039998	0.043448	45
M10	0.035745	0.040474	0.044021	28
M20	0.036045	0.0405060	0.044162	20
M30	0.036147	0.040595	0.045168	18
M40	0.036180	0.040962	0.045192	15
M50	0.037045	0.0410560	0.046011	13

**Table 7:** Dimensional stability of RPUFs made from MOAG-polyol.

Designations	Duration (Days)	Weight Changes (%)		Volume Changes (%)	
		at 70 °C	at −20 °C	at 70 °C	at −20 °C
B	1	−1.06 ± 0.03	0.49 ± 0.03	−1.19 ± 0.03	0.06 ± 0.03
	7	−1.13 ± 0.02	0.56 ± 0.03	−1.29 ± 0.04	0.57 ± 0.02
	14	−1.36 ± 0.02	0.71 ± 0.03	−1.43 ± 0.05	0.79 ± 0.03
M10	1	−0.97 ± 0.03	0.46 ± 0.03	−1.15 ± 0.05	0.11 ± 0.04
	7	−1.07 ± 0.03	0.74 ± 0.4	−1.39 ± 0.08	0.16 ± 0.03
	14	−1.18 ± 0.03	0.95 ± 0.04	−1.68 ± 0.06	0.21 ± 0.03
M20	1	−0.08 ± 0.03	0.44 ± 0.02	−0.86 ± 0.02	0.41 ± 0.04
	7	−0.95 ± 0.02	0.56 ± 0.05	−1.14 ± 0.04	0.49 ± 0.03
	14	−1.06 ± 0.01	0.67 ± 0.03	−1.17 ± 0.04	0.74 ± 0.04
M30	1	−0.96 ± 0.03	0.10 ± 0.02	−1.19 ± 0.08	0.64 ± 0.03
	7	−1.17 ± 0.04	0.16 ± 0.04	−1.25 ± 0.03	0.75 ± 0.04
	14	−1.28 ± 0.03	0.25 ± 0.03	−1.55 ± 0.03	0.95 ± 0.04
M40	1	−1.12 ± 0.03	0.75 ± 0.04	−0.06 ± 0.06	0.43 ± 0.02
	7	−1.25 ± 0.03	0.86 ± 0.02	−1.74 ± 0.04	0.56 ± 0.05
	14	−1.42 ± 0.03	0.96 ± 0.03	−1.92 ± 0.05	0.73 ± 0.03
M50	1	−0.92 ± 0.06	0.22 ± 0.02	−1.17 ± 0.02	0.07 ± 0.02
	7	−1.23 ± 0.05	0.44 ± 0.04	−1.45 ± 0.04	0.65 ± 0.03
	14	−1.31 ± 0.04	0.55 ± 0.03	−1.56 ± 0.04	0.84 ± 0.03

**Table 8:** The results of thermogravimetric analysis.

Designations	T_5%_ (°C)	T_10%_ (°C)	T_50%_ (°C)	T_max_ (°C)	Residue (%)
B	273	286	342	338	16.84
M10	270	285	348	334	18.68
M20	262	282	350	328	18.41
M30	257	280	358	325	21.63
M40	248	279	370	324	16.84
M50	237	278	375	322	17.30

## Data Availability

All data are reported in this article.
